# Effect of dual task on gait asymmetry in patients after anterior cruciate ligament reconstruction

**DOI:** 10.1038/s41598-018-30459-w

**Published:** 2018-08-13

**Authors:** Huijuan Shi, Hongshi Huang, Yuanyuan Yu, Zixuan Liang, Si Zhang, Bing Yu, Hui Liu, Yingfang Ao

**Affiliations:** 1 Institute of Sports Medicine, Peking University Third Hospital, Beijing Key Laboratory of Sports Injuries, Beijing, China; 2 Center for Human Movement Science, Division of Physical Therapy, School of Medicine, The University of North Carolina at Chapel Hill, Chapel Hill, NC USA; 3 Biomechanics Laboratory, College of Human Movement Science, Beijing Sport University, Beijing, China

## Abstract

Individuals who received anterior cruciate ligament (ACL) reconstruction surgeries demonstrated lower extremity movement asymmetries. The purpose of this study was to determine if psychological impairment was a contributor to lower extremity movement asymmetries in walking for individuals who received ACL reconstruction surgeries. Three-dimensional videographic and force plate data were collected for 25 males after unilateral ACL reconstruction performing walking without (single-task condition) and with the concurrent cognitive task (dual-task condition). Both uninjured and injured legs had significantly smaller peak knee flexion angle and peak knee extension moment during loading response and mid-stance phases in dual-task condition compared to single task condition (P ≤ 0.012). Walking condition and leg had significant interaction effects on peak hip adduction angle during mid-stance phase (P = 0.042) and peak hip abduction moment during loading response phase (P = 0.048). The inter-leg difference of peak hip adduction angle during mid-stance (P = 0.038) and terminal stance (P = 0.036) phases, and peak hip abduction moment during loading response phase (P = 0.024) were significantly decreased in dual-task condition compared to single-task condition. Psychological factors have significant effects on post-operative movements of both injured and uninjured knees of individuals who received ACL reconstruction surgery. Although physical factors may be primary contributors to the post-operative lower extremity movement asymmetries, psychological factors also contribute to the post-operative hip movement asymmetries.

## Introduction

Anterior cruciate ligament (ACL) reconstruction is a commonly surgical procedure to restore knee functions after an ACL injury^1^. Approximately 175000 ACL reconstruction surgeries were performed in the United States annually^2^. Although ACL reconstruction surgeries restored knee stability, studies repeatedly reported high ACL re-injury rates and early development of knee osteoarthritis (OA) after ACL reconstruction. Literatures showed that up 12% of patients with reconstructed ACLs had ACL re-injury^3,4^ and the prevalence of radiographic knee OA of patients with combined ACL and meniscal injuries in 10 to 15 years follow-up after ACL reconstruction were 80%^5^.

Lower extremity movement asymmetries were found to be predictive of ACL re-injury and knee OA after ACL reconstruction^6–8^. Literature showed that athletes with multiplane biomechanical asymmetries at hip and knee at the time of return to sport were at least three times more likely to have ACL re-injury within one year compared to those without these asymmetries^7^. Literature also showed that asymmetrical lower extremity loading altered chondrocyte synthesis and catabolic activities, which could lead to structural damage of articular cartilage and may accelerate the development of knee OA^9–11^.

Weak quadriceps strength was identified as a primary contributor to lower extremity movement asymmetries after ACL reconstruction surgery. Studies showed that impaired quadriceps strength was correlated to altered movement patterns of injured leg after ACL reconstruction surgery^12,13^. Patients with quadriceps strength asymmetry demonstrated increased asymmetry in lower extremity kinetics in functional activities compared to patients with quadriceps strength symmetry^14,15^. These studies appear to indicate impaired quadriceps strength was the contributor to lower extremity movement asymmetries after ACL reconstruction. A study by Roewer *et al*., however, showed that asymmetric knee angles, knee moments, and hip power still existed despite subjects achieving symmetric quadriceps strength after ACL reconstruction^16^. Lentz *et al*. also reported that despite quadriceps strength was improved from 6 months to 1 year, functional limitations remained unchanged^17^. These studies indicated that quadriceps strength deficit was not the only contributor to lower extremity movement asymmetries.

In addition to physical impairments, psychological impairments could also contribute to lower extremity movement asymmetries after ACL reconstruction. Movement asymmetries after ACL reconstruction surgery may be due to patients’ preference of using their uninjured leg, which could put the contralateral leg at high risk for injury^18^. A meta-analysis of the state of play revealed that fear for re-injury was the most common reason for post-operative reduction in or cessation of sports participation^19^. A study on psychological changes of patients who received ACL reconstruction surgery found that patients who returned to the competitive sport at 12 months after the surgery had more positive psychological responses to sports participation at 6 and 12 months after the surgery compared to patients who had not returned to competitive sport^20^. Although these studies indicated that psychological impairments may contribute to patients’ movement asymmetries after ACL reconstruction surgery, the effect of psychological impairment on lower extremity movement asymmetries has not been confirmed yet.

The purpose of this study was to determine if psychological impairment was a contributor to lower extremity movement asymmetries in walking for individuals who received ACL reconstruction surgeries. We hypothesized that there would be a quadriceps strength asymmetry after ACL reconstruction surgery. We also hypothesized that there would be asymmetries in hip and knee angles and moments in walking after ACL reconstruction surgery. We further hypothesized that asymmetries of hip and knee angles and moments would be decreased when walking with a cognitive task compared to walking without the cognitive task.

## Methods

### Participants

Twenty-five males who received unilateral ACL reconstruction using hamstring tendon autograft within 10 months before the study (age = 32 ± 8.2 years, height = 1.75 ± 0.07 m, weight = 84.9 ± 12.3 kg, 7.4 ± 1.3 months past reconstruction) volunteered to participate in this study. All participants had isolated ACL injury without other ligament and meniscus injuries that needed to be repaired with a resection or suture, or moderate or severe articular cartilage damage to the patellofemoral and tibiofemoral joint, or other current orthopaedic injuries or disorders that were still affecting lower extremity movements. All participants went through similar post-operative home-based rehabilitation programs and had normal knee range of motion at the time of participating in the study. The mean International Knee Documentation Committee (IKDC) score of the participants was 67.4 ± 11.4. The use of human subjects in this study was approved by the Institutional Review Board of Peking University Third Hospital. All participants read and signed a written informed consent before data collection.

### Protocol

Each participant was asked to wear a pair of black spandex shorts. Passive reflective markers were placed bilaterally at the anterior superior iliac spine, posterior superior iliac spine, lateral thigh, lateral femoral condyle, medial femoral condyle, anterior superior shank, anterior inferior shank, lateral malleoli, medial malleoli, heel, and first and fifth metatarsophalangeal. The participant was instructed to walk with barefoot in two conditions: (1) without cognitive task (single-task condition), and (2) with concurrent cognitive task (dual-task condition). In single-task condition, the participant walked along a 10-m walkway at self-selected walking speed. In dual-task condition, the participant walked along the walkway while backward counting numbers with an increment of seven starting from a randomly given number between 125 and 250. In dual-task condition, participants immediately initiated the walking task at their preferred speed once they heard the number read by the investigator and kept counting out loudly as required without repeating the given number. Participants were asked to count as fast as possible while walking task. A 5 min rest was requested between single-task and dual-task test. The order of conditions was randomized for the participant. Quadriceps strength was evaluated for the participant after walking test.

### Data Collection

Three-dimensional (3-D) trajectories of the reflective markers were collected using an 8-camera motion capture system (VICON, Oxford, UK) at a sample rate of 100 Hz. Ground-reaction force signals were collected using two embedded force plates (AMTI, Watertown, Massachusetts) at a sample rate of 1000 Hz. Each participant was asked to have three successful trials for each condition. A successful trial was defined as a trial in which the participant performed the task as required, and all kinematic and kinetic data were collected.

Quadriceps isometric strength was quantified with an isokinetic dynamometer (CON-TREX MJ; Germany) during a maximum voluntary isometric contraction (MVIC). The participant was seated with a hip flexed at 90° and knee flexed at 60°. The lateral femoral condyle was aligned with the dynamometer axis, and the dynamometer resistance pad was secured to the anterior aspect of the distal shank. After correcting for leg weight, the participant was asked to perform submaximal practice to familiarize themselves with the testing apparatus. After familiarization of testing apparatus, the participant was asked to have three recorded maximum-effort trials (5 seconds in duration, 60 seconds’ rest between trials) for each leg with uninjured leg tested first.

### Data Reduction

The raw 3-D trajectories of the reflective markers were filtered through a Butterworth low-pass digital filter at an estimated optional cut-off frequency of 10 Hz^21^. Kinematics and kinetics variables were calculated using Visual 3D software (C-Motion Inc., Germantown, Maryland, USA). Hip and knee joint angles were calculated as Cardan angles of distal segment reference frame relative to the proximal segment reference frame in an order of (1) flexion-extension, (2) abduction-adduction, and (3) internal-external rotation. Joint moments were calculated through an inverse dynamic approach and transferred into the distal segment reference frame. Joint moments and quadriceps strength were normalized to the production of body weight and standing height.

**Figure 1:**
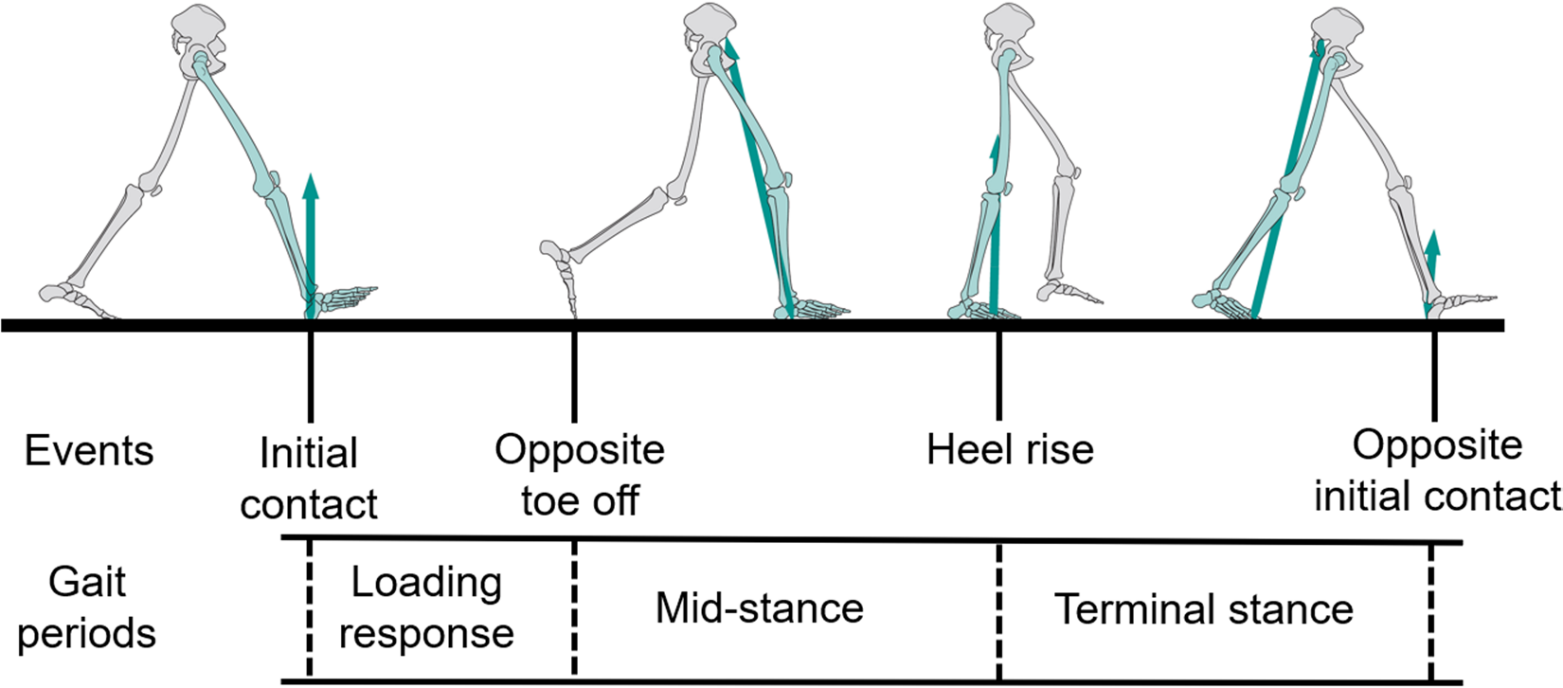
Three phases during walking. Four major events (Initial contact, opposite toe off, heel rise, opposite initial contact) subdivide corresponding phase into three periods (Loading response phase, mid-stance phase, and terminal stance phase) in this study.

Three phases, loading response phase, mid-stance phase, and terminal stance phase, during walking were analyzed (Fig. 1). Knee range of motion, hip range of motion, peak hip adduction angle, peak hip and knee abduction moment during loading response phase, mid-stance phase, and terminal stance phase, peak knee flexion angle, peak knee extension moment, peak knee adduction angle during loading response and mid-stance phases, minimum hip flexion angle, peak hip extension moment during loading response phase, peak hip flexion moment during mid-stance and terminal stance phases, peak hip extension angle and peak knee flexion moment during terminal stance phase, and walking speed were identified for each subject in each trial. Walking speed was calculated using

$$Walking\,Speed={L}_{stridelength}/{T}_{cycletime}$$

where *L*_*stride length*_ was the forward distance of heel marker between successive heel contacts of the same foot, *T*_*cycle time*_ was the time between the first contacts of 2 consecutive footfalls of the same foot.

The inter-leg difference (ILD) between injured and uninjured legs was also calculated for joint angles and moments as described in literature^22^

$$ILD={Y}_{uninjuredleg}-{Y}_{injuredleg}$$

where *Y*_*uninjured leg*_ and *Y*_*injured leg*_ were magnitudes of a given joint angle or moment of uninjured leg and injured leg.

### Data Analysis

Paired t-test was performed to compare the walking speed between single-task and dual-task conditions and isometric quadriceps strength between injured and uninjured legs. Two-way analyses of variance (ANOVA) with repeated measures were performed to compare the kinematic and kinetic variables of interest between conditions (single-task and dual-task conditions) and legs (injured leg and uninjured leg). In case a significant interaction effect of condition and leg was detected, paired t-tests were performed to compare the dependent variables between legs in a given condition and between conditions on a given leg. Paired t-tests were performed to compare the ILD between single-task and dual-task conditions. All statistical analyses were performed using SPSS computer program package, version 16.0 (SPSS Inc., Chicago, IL, USA). A type I error rate less than or equal to 0.05 was considered as the indication of statistical significance.

## Results

Walking speed in dual task condition (1.15 ± 0.13 m/s) was significantly lower than that in single task condition (1.24 ± 0.14 m/s) (P = 0.003). The quadriceps strength of injured leg (0.096 ± 0.028 BW × BH) was significantly smaller than that of uninjured leg (0.129 ± 0.028 BW × BH) (P = 0.001).

No significant interaction effect of condition and leg on knee angular kinematics were detected (P ≥ 0.162). Injured leg had smaller peak knee flexion angle during loading response and mid-stance phases compared to uninjured leg (P = 0.004, P = 0.005) (Table 1). Injured leg also had smaller knee range of motion during mid-stance and terminal stance phases compared to uninjured leg (P = 0.001, P = 0.001) (Table 1). Both uninjured and injured legs had significantly smaller peak knee flexion angle during loading response and mid-stance phases in dual-task condition compared to single task condition (P = 0.002, P = 0.009) (Table 1). Also, both uninjured and injured legs had significantly smaller knee range of motion during loading response and mid-stance phases in dual-task condition compared to single task condition (P = 0.006, P = 0.002) (Table 1). ILD for knee range of motion during terminal stance phase was significantly increased in dual-task condition compared to single-task condition (P = 0.017).

**Table 1:** Knee angles (mean ± standard deviation) (degrees) and inter-leg difference (ILD) of injured and uninjured leg.

Variable	Leg	Single-task	Dual-task	P*-*value
Peak knee flexion angle during loading response phase	Uninjured	16.4 ± 8.1	15.4 ± 7.7	0.002
	Injured	13.4 ± 6.7	11.9 ± 7.1	
	P-value	0.004		
	ILD	3.0 ± 5.2	3.5 ± 5.1	0.396
Knee range of motion during loading response phase	Uninjured	11.7 ± 4.5	10.8 ± 4.1	0.006
	Injured	10.2 ± 4.3	9.1 ± 3.8	
	P-value	0.053		
	ILD	1.5 ± 4.1	1.7 ± 3.9	0.302
Peak knee adduction angle during loading response phase	Uninjured	1.1 ± 1.9	1.0 ± 1.9	0.802
	Injured	1.3 ± 1.9	1.5 ± 2.4	
	P-value	0.383		
	ILD	−0.1 ± 1.9	−0.5 ± 1.8	0.181
Peak knee flexion angle during mid-stance phase	Uninjured	16.8 ± 8.0	15.6 ± 7.7	0.009
	Injured	13.6 ± 6.9	12.2 ± 7.3	
	P-value	0.005		
	ILD	3.1 ± 5.3	3.4 ± 5.4	0.405
Knee range of motion during mid-stance phase	Uninjured	10.2 ± 4.4	9.3 ± 4.7	0.002
	Injured	6.7 ± 3.1	5.4 ± 2.9	
	P-value	0.001		
	ILD	3.5 ± 3.7	3.8 ± 4.5	0.442
Peak knee adduction angle during mid-stance phase	Uninjured	1.1 ± 2.0	1.1 ± 1.8	0.745
	Injured	1.2 ± 2.2	1.2 ± 2.3	
	P-value	0.668		
	ILD	−0.2 ± 2.0	−0.2 ± 1.8	0.354
Minimum knee flexion angle during terminal stance phase	Uninjured	5.2 ± 4.4	5.3 ± 4.7	0.916
	Injured	6.4 ± 5.0	6.3 ± 5.9	
	P-value	0.254		
	ILD	−1.2 ± 4.1	−0.9 ± 4.9	0.306
Knee range of motion during terminal stance phase	Uninjured	7.5 ± 2.0	7.5 ± 2.1	0.139
	Injured	5.5 ± 2.1	5.0 ± 2.5	
	P-value	0.001		
	ILD	2.0 ± 1.9	2.5 ± 2.0	0.017

No significant interaction effects of condition and leg on knee moments were detected (P ≥ 0.193). Injured leg had smaller peak knee flexion moment during terminal stance phase compared to uninjured leg (P = 0.001) (Table 2). Both uninjured and injured legs had significantly smaller peak knee extension moment during loading response and mid-stance phases in dual-task condition compared to single-task condition (P = 0.012, P = 0.001) (Table 2). Also, both uninjured and injured legs had significantly smaller peak knee abduction moment during mid-stance phase in dual-task condition compared to single-task condition (P = 0.001) (Table 2).

**Table 2:** Knee moments (mean ± standard deviation) (BW * BH * 10^−2^) and inter-leg difference (ILD) of injured and uninjured leg.

Variable	Leg	Single-task	Dual-task	P-value
Peak knee extension moment during loading response phase	Uninjured	3.26 ± 1.45	2.95 ± 1.26	0.012
	Injured	2.75 ± 1.13	2.59 ± 0.95	
	P-value	0.060		
	ILD	0.51 ± 1.21	0.36 ± 1.02	0.482
Peak knee adduction moment during loading response phase	Uninjured	1.00 ± 0.94	0.94 ± 0.95	0.614
	Injured	0.93 ± 0.79	0.91 ± 0.86	
	P-value	0.798		
	ILD	0.06 ± 1.14	0.03 ± 0.96	0.138
Peak knee abduction moment during loading response phase	Uninjured	2.14 ± 0.54	2.04 ± 0.56	0.155
	Injured	1.87 ± 0.44	1.82 ± 0.58	
	P-value	0.093		
	ILD	0.27 ± 0.69	0.22 ± 0.71	0.434
Peak knee extension moment during mid-stance phase	Uninjured	3.32 ± 1.52	2.91 ± 1.35	0.001
	Injured	2.92 ± 1.34	2.57 ± 1.35	
	P-value	0.130		
	ILD	0.40 ± 1.11	0.34 ± 1.27	0.120
Peak knee abduction moment during mid-stance phase	Uninjured	2.32 ± 0.54	2.20 ± 0.49	0.001
	Injured	2.11 ± 0.52	1.97 ± 0.58	
	P-value	0.134		
	ILD	0.21 ± 0.76	0.23 ± 0.69	0.187
Peak knee flexion moment during terminal stance phase	Uninjured	1.15 ± 0.75	1.04 ± 0.68	0.170
	Injured	0.31 ± 0.67	0.32 ± 0.80	
	P-value	0.001		
	ILD	0.84 ± 0.84	0.71 ± 0.91	0.226
Peak knee abduction moment during terminal stance phase	Uninjured	2.10 ± 0.70	1.99 ± 0.64	0.060
	Injured	1.83 ± 0.72	1.76 ± 0.72	
	P-value	0.117		
	ILD	0.27 ± 0.80	0.23 ± 0.75	0.232

A significant interaction effect of condition and leg on peak hip adduction angle during mid-stance phase was detected (P = 0.042). Injured leg had significant smaller peak hip adduction angle during mid-stance phase compared to uninjured leg in single-task condition (P = 0.050) while no significant difference was found in this variable between legs in dual-task condition (P = 0.256) (Table 3). Both uninjured and injured legs had significantly smaller peak hip adduction angle during mid-stance phase in dual-task condition compared to single-task condition (P = 0.001, P = 0.009) (Table 3). ILD for peak hip adduction angle during mid-stance phase was significantly decreased in dual-task condition compared to single-task condition (P = 0.038) (Table 3).

**Table 3:** Hip angles (mean ± standard deviation) (degrees) and inter-leg difference (ILD) of injured and uninjured leg.

Variable	Leg	Single-task	Dual-task	P-value
Minimum hip flexion angle during loading response phase	Uninjured	21.9 ± 6.3	20.7 ± 5.8	0.002
	Injured	20.0 ± 5.6	18.6 ± 5.4	
	P-value	0.004		
	ILD	1.9 ± 3.0	2.1 ± 3.5	0.153
Hip range of motion during loading response phase	Uninjured	4.2 ± 2.3	4.8 ± 2.4	0.032
	Injured	5.3 ± 2.7	5.7 ± 2.8	
	P-value	0.031		
	ILD	−1.0 ± 2.2	−0.9 ± 2.2	0.162
Peak hip adduction angle during loading response phase	Uninjured	8.2 ± 2.9	7.4 ± 2.7	0.003
	Injured	7.1 ± 3.6	6.6 ± 3.5	
	P-value	0.152		
	ILD	1.1 ± 3.3	0.8 ± 3.1	0.239
Hip range of motion during mid-stance phase	Uninjured	23.1 ± 3.7	21.4 ± 4.4	0.001
	Injured	22.1 ± 3.2	20.1 ± 3.7	
	P-value	0.083		
	ILD	1.1 ± 3.1	1.3 ± 4.0	0.412
Peak hip adduction angle during mid-stance phase	Uninjured	9.1 ± 2.6	8.0 ± 2.5	<0.001
	Injured	7.8 ± 3.5	7.2 ± 3.5	0.009
	P-value	0.050	0.256	
	ILD	1.2 ± 3.1	0.7 ± 3.1	0.038
Peak hip extension angle during terminal stance phase	Uninjured	12.3 ± 4.5	11.7 ± 4.8	0.010
	Injured	13.6 ± 4.3	13.1 ± 4.9	
	P-value	0.034		
	ILD	−1.3 ± 2.6	−1.4 ± 3.4	0.115
Hip range of motion during terminal stance phase	Uninjured	11.0 ± 2.3	11.1 ± 1.7	0.928
	Injured	11.5 ± 2.6	11.6 ± 3.3	
	P-value	0.384		
	ILD	−0.5 ± 2.9	−0.5 ± 3.1	0.345
Peak hip adduction angle during terminal stance phase	Uninjured	6.8 ± 2.3	6.3 ± 2.3	0.037
	Injured	5.6 ± 2.5	5.5 ± 2.6	
	P-value	0.050		
	ILD	1.2 ± 2.3	0.8 ± 2.5	0.036

No significant interaction effects of condition and leg on other hip angular kinematics were detected (P ≥ 0.055). Injured leg had smaller minimum hip flexion angle during loading response phase compared to uninjured leg (P = 0.004) (Table 3). Injured leg had greater hip range of motion during loading response phase compared to uninjured leg (P = 0.031) (Table 3). Injured leg had greater hip extension angle during terminal stance phase compared to uninjured leg (P = 0.034) (Table 3). Injured leg also had smaller peak hip adduction angle during terminal stance phase compared to uninjured leg (P = 0.050) (Table 3). Both uninjured and injured legs had significantly smaller minimum hip flexion angle during loading response phase in dual-task condition compared to single task condition (P = 0.002) (Table 3). Also, both uninjured and injured legs had significantly smaller hip range of motion during loading response and mid-stance phases in dual-task condition compared to single task condition (P = 0.032, P = 0.001) (Table 3). Similarly, both uninjured and injured legs had significantly smaller peak hip adduction angle during loading response and terminal stance phases in dual-task condition compared to single task condition (P = 0.003, P = 0.037) (Table 3). Both uninjured and injured legs had significantly smaller peak hip extension angle during terminal stance phase in dual-task condition compared to single task condition (P = 0.010) (Table 3). ILD for peak hip adduction angle during terminal phase was significantly decreased in dual-task condition compared to single-task condition (P = 0.036).

A significant interaction effect of condition and leg on peak hip abduction moment during loading response phase was detected (P = 0.048). Injured leg had significantly smaller peak hip abduction moment during loading response phase compared to uninjured leg in both single task and dual task condition (P = 0.034, P = 0.004) (Table 4). Uninjured leg had significant smaller peak hip abduction moment during loading response phase in dual-task condition compared to single-task condition (P = 0.009) while injured leg had no significant difference in this variable between conditions (P = 0.159) (Table 4). The ILD for peak hip abduction moment during loading response phase was significantly decreased in dual-task condition compared to single-task condition (P = 0.024).

**Table 4:** Hip moments (mean ± standard deviation) (BW*BH*10^−2^) and inter-leg difference (ILD) of injured and uninjured leg.

Variable	Leg	Single-task	Dual-task	P-value
Peak hip extension moment during loading response phase	Uninjured	7.21 ± 3.38	6.52 ± 2.99	0.045
	Injured	6.91 ± 2.79	6.40 ± 3.05	
	P-value	0.660		
	ILD	0.29 ± 2.67	0.12 ± 2.19	0.357
Peak hip adduction moment during loading response phase	Uninjured	1.68 ± 1.43	1.59 ± 1.60	0.860
	Injured	1.27 ± 0.96	1.31 ± 1.15	
	P-value	0.228		
	ILD	0.40 ± 1.60	0.30 ± 1.29	0.408
Peak hip abduction moment during loading response phase	Uninjured	4.88 ± 0.96	4.56 ± 1.26	0.009
	Injured	4.08 ± 0.83	3.94 ± 0.85	0.159
	P-value	0.032	0.004	
	ILD	0.80 ± 1.22	0.62 ± 1.33	0.024
Peak hip flexion moment during mid-stance phase	Uninjured	1.31 ± 0.69	1.23 ± 0.66	0.332
	Injured	1.39 ± 0.74	1.29 ± 0.80	
	P-value	0.500		
	ILD	−0.08 ± 0.66	−0.06 ± 0.52	0.094
Peak hip abduction moment during mid-stance phase	Uninjured	5.16 ± 0.65	4.83 ± 0.64	0.001
	Injured	4.73 ± 0.65	4.47 ± 0.72	
	P-value	0.023		
	ILD	0.43 ± 0.78	0.36 ± 0.91	0.305
Peak hip flexion moment during terminal stance phase	Uninjured	4.67 ± 0.94	4.37 ± 1.09	0.001
	Injured	4.59 ± 1.11	4.14 ± 1.11	
	P-value	0.213		
	ILD	0.07 ± 0.78	0.23 ± 0.54	0.078
Peak hip abduction moment during terminal stance phase	Uninjured	4.90 ± 0.54	4.86 ± 0.38	0.441
	Injured	4.72 ± 0.72	4.67 ± 0.64	
	P-value	0.171		
	ILD	0.18 ± 0.71	0.19 ± 0.70	0.451

No significant interaction effects of condition and leg on other hip moments were detected (P ≥ 0.214). Injured leg had smaller peak hip abduction moment during mid-stance phase compared to uninjured leg (P = 0.023) (Table 4). Both uninjured and injured legs had significantly smaller peak hip extension moment during loading response phase in dual-task condition compared to single task condition (P = 0.045) (Table 4). Also, both uninjured and injured legs had significantly smaller peak hip flexion moment during terminal stance phase in dual-task condition compared to single task condition (P = 0.001) (Table 4). Both uninjured and injured legs had significantly smaller peak hip abduction moment during mid-stance phase in dual-task condition compared to single task condition (P = 0.001) (Table 4). No difference was found in ILD for other hip moments between single-task and dual-task condition (P ≥ 0.078) (Table 4).

## Discussion

The results of this study support our hypothesis that there would be a quadriceps strength asymmetry after ACL reconstruction surgery. The decreased quadriceps strength of injured leg in this study was consistent with the quadriceps strength asymmetries reported in literatures^22–24^. These results of this study combined with the literatures indicated that participants in this study indeed had quadriceps asymmetries as defined in literatures. A previous study demonstrated that increased quadriceps strength was associated with increased knee cartilage and greater cartilage cross-sectional area^25^, indicating quadriceps weakness as a possible contributor to the development of knee osteoarthritis of individuals received ACL reconstruction surgeries. Extended rehabilitation programs, therefore, are needed to correct quadriceps strength asymmetry after ACL reconstruction surgery to minimize the risk of early knee osteoarthritis.

The results of this study support our hypothesis that there would be asymmetries in hip and knee motion patterns in walking after ACL reconstruction surgery. The decreased peak knee flexion angle during the loading response phase of injured leg observed in this study is consistent with literatures^26–28^. These results suggested that individuals who received ACL reconstruction surgeries tended to walk with increased leg stiffness^29^. The results of this study further showed that injured leg had smaller peak knee flexion moment during terminal stance phase while having no significant difference in peak knee abduction moments during mid-stance and terminal stance phases between injured and uninjured legs, which is also consisitent with literature^30,31^. Literatures show that knee moment in the sagittal plane is positively correlated with medial femoral cartilage thickness in healthy individuals^32^, and that approximately 85% of the mechanical work during gait is done in the sagittal plane^33^. The sagittal-plane moments appears to be more revelant to the knee joint degeneration after ACL reconstruction surgery than peak knee abduction moment. The results of current study also showed that injured leg had smaller peak hip abduction moment during the mid-stance phase compared to uninjured leg while having no significant difference in peak hip abduction moment during terminal stance phase. The decreased peak hip abduction moment in the mid-stance phase in this study was likely due to weaker hip abductors. Hip abductors assist in stabilizing pelvis in the frontal plane. Ipsilateral hip abductor weakness may cause the pelvis to drop towards the contralateral swing leg^34^. A recent study showed that patients who develop medial compartment knee OA within 5 years after ACL reconstruction surgery walked with asymmetrically lower peak hip abduction moments compared to those who did not develop knee OA at 5 years^35^. These results combined together suggested that altered hip movement patterns in the frontal plane may be a vital predictor to initiate knee degeneration and may need to be addressed during rehabilitation.

The results of this study partially support our hypothesis that asymmetries of hip and knee angular kinematics and moments would be decreased when walking with a cognitive task compared to walking without the cognitive task. Interaction effects of condition and leg on hip adduction angles and abduction moments and decreased corresponding ILDs observed this study suggested that asymmetries of hip movements in the frontal plane was decreased while walking in dual task condition. No similar results were obtained for knee angles and moments. Baumeister *et al*. reported that patients after ACL reconstruction surgery increased brain activation in attentional and sensory areas during force reproduction task compared to the healthy control group, which reflect an increased focused attention and therefore an increased neurocognitive involvement^36^. According to the limited capacity theory of attention, when an attention-demanding cognitive task imposed on an attention-demanding task, concurrent execution of both tasks may require more attentional capacity than the existing capacity and therefore one or both tasks could not have sufficient attention^37^. Walking while performing a cognitive task decreased attention distribution to walking in the dual task condition compared to single task condition in this study. In this situation, the decreased ILD should be considered as a result of decreased psychological effects on asymmetries of walking. These results, therefore, indicate that psychological as well as physical factors contribute to movement asymmetries post ACL reconstruction surgeries.

Although the asymmetries of knee angular kinematics and moments were not decreased while walking with a cognitive task, post-operative knee movements of patients who received ACL reconstruction surgery were still affected by psychological factors. The results of this study showed that participants significantly decreased their knee angles and moments while walking with a cognitive task. A study showed that healthy individuals prioritized gait control at the cost of performance of the cognitive task in dual task condition, while patients with Parkinson’s disease prioritized performance of the cognitive task at the cost of performance of gait^38^. Considering these results, although we don’t know the cost of the performance of the cognitive task when participants walking in dual task condition in current study, the results of current study indicated that performing cognitive task while walking was indeed at the cost of the performance of gait for individuals who received ACL reconstruction surgery, which is similar to that of patients with Parkinson’s disease, but different from that of healthy individuals. This means that psychological factors still affect post-operative gait for individuals who received ACL reconstruction surgeries, but this effect may be similar for both injured and uninjured legs.

Psychological factors might have different influence on post-operative hip and knee movement patterns in walking for individuals who received ACL reconstruction surgeries. The results of this study showed that ILDs of hip angular kinematics and moments in the frontal plane were decreased in dual task condition compared to single task condition, while ILDs of knee angular kinematics and moments were essentially the same between conditions. These results indicated that effects of psychological factors on asymmetries of hip and knee movements are different. The hip joint is the most proximal joint in the lower extremity kinematic chain and shares the femur with the knee joint. Winter *et al*. suggested that postural stability in the frontal plane was controlled primarily by activation of the hip abductors and adductors designed to alternately load and unload the hips, thereby controlling the movement of center of mass^39^. Abnormal hip movements could have a deleterious effect on the tibiofemoral and patellofemoral mechanics in multiple planes^40^. This means that the effects of psychological factors on lower extremity movements could be mainly through their effects on the movements of hip joints. Interventions which address proximal movement impairments may be beneficial for patients who had various knee movement impairments. As participants had lower quadriceps strength of injured leg compared to uninjured leg in this study, their knee movements might be mainly affected by physical factors while hip movements affected by both physical as well as psychological factors.

Our findings have important implications for post ACL reconstruction rehabilitation programs. The risk of ACL re-injury remains higher than that of the initial ACL injury^41^, likely due to either poor movement strategies or lingering impairments that were not addressed by surgical reconstruction and post-surgery rehabilitation. Movement strategies are modifiable and represent important targets for successful clinical interventions. The results of this study indicated that psychological factors are contributors to the post-operative lower extremity movement asymmetries of individuals who received ACL reconstruction surgeries. As psychological differences regarding returning to sports among athletes who received surgeries treating injuries occur as early as six months postoperatively^20^. Clinicians should address psychological factors early to help patients modify the abnormal movement patterns and facilitate their returns to competitive sport. Though the results of this study indicated that the immediate effect of the secondary cognitive task did not decrease the asymmetry of knee movements, the asymmetry of hip movements in frontal plane could be modified in dual-task condition. Dual-task training could be used as an intervention to improve movement performance and motor control in rehabilitation programs^42^.

Several limitations of this study may need to be addressed in the future studies. Since the asymmetry of quadriceps strength could affect the lower extremity movement symmetry^14,15^, the effect of the concurrent cognitive task on walking may be different between the group with symmetrical quadriceps strength and the group with asymmetrical quadriceps strength. Future studies may be needed to assess the effects of physical factors on the effects psychological factors on lower extremity movement asymmetries. In addition, since gender differences in lower extremity movement patterns exist^43^, psychological factors may affect lower extremity movement asymmetries differently between genders. Future studies should include both females and males to investigate the gender differences in psychology.

## Conclusions

The results of this study appear to warrant the following conclusions:

1. There were significant post-operative asymmetries in quadriceps strength, and hip and knee movements of individuals who received ACL reconstruction surgery.
2. Psychological factors have significant effects on post-operative movements of both injured and uninjured knees of individuals who received ACL reconstruction surgery.
3. Although physical factors may be primary contributors to the post-operative lower extremity movement asymmetries, psychological factors also contribute to the post-operative hip movement asymmetries.
